# Deciphering the Dialogue between Brain Tumors, Neurons, and Astrocytes

**DOI:** 10.1016/j.ajpath.2025.04.013

**Published:** 2025-05-07

**Authors:** Leevi H. Westerlund, Camilla K. Bergström, Pirjo M. Laakkonen, Vadim Le Joncour

**Affiliations:** ∗ Translational Cancer Medicine Research Program–CAN-PRO, Faculty of Medicine, University of Helsinki, Helsinki, Finland; † Helsinki University Central Hospital, Helsinki, Finland; ‡ Neuroscience Center, HiLIFE–Helsinki Institute of Life Science, University of Helsinki, Helsinki, Finland; § iCAN Digital Precision Cancer Medicine Flagship Program, University of Helsinki and Helsinki University Hospital, Helsinki, Finland; ¶ Laboratory Animal Centre, HiLIFE–Helsinki Institute of Life Science, University of Helsinki, Helsinki, Finland

## Abstract

Glioblastoma and brain metastases from peripheral tumors account for most cases of tumors in the central nervous system while also being the deadliest. From a structural point of view, malignant brain tumors are classically characterized by hypercellularity of glioma and vascular endothelial cells. Given these atypical histologic features, glioblastoma and brain metastases have long been considered as “foreign” entities with few to no connections to the brain parenchyma. The identification of intricate connections established between glioblastoma cells and the brain parenchyma paired with the ability of peripheral metastatic cells to form functional synapses with neurons challenged the concept of brain tumors disconnected from the central nervous system. Tumor cell integration to the brain parenchyma alters brain functionality in patients and accelerates cancer progression. Next-generation precision medicine should therefore attempt to disconnect brain cancer cells from the brain. This review encompasses recent discoveries in the mechanisms underlying these relationships and discusses the impact of these connections on tumor progression. It also summarizes the therapeutic opportunities of interrupting the dialogue between healthy and neoplastic brains.

## Graphical abstract

**Figure:**
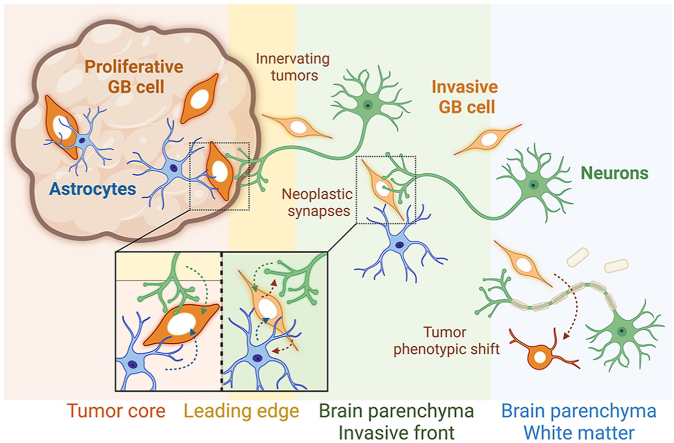

Cancers of the central nervous system (CNS) strike blindly and are frequently associated with a dismal prognosis. Primary brain tumors (malignant and nonmalignant) affect women more than men (26.31 versus 21.09 per 100,000). However, malignant tumors are more common in men (56% of the cases). Primary brain tumors are also the leading cause of childhood cancer–related deaths.^1^ Glioblastoma (GB) is the most aggressive primary brain cancer, accounting for 14.5% of all primary brain tumors alone. Brain metastases (BM) are even more frequent, as they occur in about 40% of patients with metastatic cancer and represent up to 80% of all cases of intracranial tumors.^2^

Current therapeutic protocol is decided based on the histopathologic diagnosis and is adjusted according to the tumor size, location in the brain, and patient’s age and health.^3^ For GB, the conventional approach is surgical resection followed by antimitotic treatments via large-field or locoregional radiotherapy supplemented by cycles of the DNA-damaging drug temozolomide.^4^ BM occur at an advanced stage of primary cancer progression, and surgical craniotomy increases the burden of weaker patients. Clinicians can opt for noninvasive, stereotactic gamma-knife intervention, consisting of narrow-beam radiation of the patient’s head targeted to the identified metastatic sites.^2^ However, progression of BM is hard to impede and, in the absence of effective treatments, shortly leads to death.

Nomenclature indicates that GB primarily originates from a glial lineage. However, histologic features of GB do not consistently recapitulate those observed for healthy glial cell networks. GBs often feature hypercellularity and a highly active metabolism leading to hypoxic/necrotic areas. This chronic oxygen deficit triggers uncontrolled angiogenic growth.^5^ The resulting enlarged, leaky, fibrotic, and hemorrhagic tumor blood vessels do not share the proper structure of functional brain blood vessels. In addition to engineering *de novo* tumor blood vessels, GB cells use preexisting brain endothelial capillaries as a scaffold for invasion.^6^ This enables a rapid tumor invasion to distant brain areas, faster than with other cellular scaffolds such as white matter tracts.^7^

At the inception of cancer neuroscience, it was suspected that neoplastic cells used molecular and physical features from distinct cerebral areas (eg, the corpus callosum) to invade and grow. Tumor cell interactions were mainly based on the composition of the microenvironment but not necessarily local brain cell populations.^7^ More recent discoveries changed this preconception of a passive tumor growth devoid of active connection to the brain parenchyma. For instance, GB^8^ and BM^9^ cells can form their own electrically active glutamatergic synapses with neurons (Table 1^8^, ^9^, ^10^, ^11^, ^12^, ^13^, ^14^, ^15^, ^16^, ^17^, ^18^, ^19^, ^20^, ^21^, ^22^, ^23^, ^24^, ^25^, ^26^, ^27^, ^28^, ^29^, ^30^, ^31^, ^32^, ^33^, ^34^, ^35^, ^36^, ^37^, ^38^, ^39^, ^40^, ^41^, ^42^, ^43^, ^44^, ^45^, ^46^, ^47^, ^48^, ^49^, ^50^, ^51^, ^52^, ^53^, ^54^, ^55^, ^56^, ^57^, ^58^, ^59^, ^60^, ^61^). These provocative findings, showing that cancer cells from very different lineages can “speak” the same language as native neurons, also revealed how these neoplastic synapses accelerated progression and resistance to therapies.^8^^,^^9^ These first discoveries on the interplay between brain tumors and CNS were made less than a decade ago. In 2025, two ongoing clinical trials are attempting to pharmacologically sever brain tumors from neuronal and astrocyte networks. Using neuroscientific tools, these efforts are potentially paving the way for next-generation precision medicine targeting GB and BM.

**Table 1:** Interactome Used in the Crosstalk between Malignant Brain Tumor Cells (Primary and Brain Metastases), Astrocytes, and Neurons

Element of language	Source	Paracrine or physical connexion	Receiver	Pro- or *antitumoral* biological activity	Organism	Model	Therapeutic application	Reference
AMPA	GB	**Microtubes**	Neurons	Migration, proliferation	Hu	PDX, patient	Isoflurane, perampanel	8
GluN2B/NMDAR	BrBM	**Synapses**		Brain colonization	Hu	PDX, patient	NA	9
CXCL5	Astrocytes	Ligand	GB	MES transition, migration, proliferation	Ms, Hu	Co-culture, allograft	NA	10
POSTN, SRGN		**Reactivity**	LGG/GB	Proliferation, increased “astrocyte signature score”	Hu	*In silico*, co-culture, PDX, patient	NA	11,12
CHI3L1/IL-13RA2		Ligand	GB	Migration, proliferation	Hu	Co-culture, patient	NA	13
MMP14/2		**Tumor ECM**			Hu	Co-culture	NA	14
IL-6		Ligand						
MGMT mRNA, miR-19a		**EVs**		Resistance to TMZ, invasiveness	Hu	*In vitro*, PDX	NA	Reviewed in 15
miR-1238	GB		Astrocytes	Invasiveness				
IL-1β	Astrocytes	Ligand	GB	MES transition, therapy resistance	Hu	Patient	Potentiate immunotherapies	16
TNF-α		Ligand						
STAT3		**Tumor ECM**						
NF-κB		Ligand						
EMT		**Reactivity**		MES transition, tumor progression	Ms, Hu	*In silico*, PDX, patient	NA	Reviewed in 17,18
	GB	**EVs**	Astrocytes					
Ion channels and transporters	Astrocytes	K^+^, Cl^–^, Ca2^+^, Na^+^	GB	Invasiveness, proliferation	Hu	In silico, *in vitro*, PDX	Ion channels blockers (psalmotoxin-1, benzamil, ouabin, digoxin, cholotoxin)	19,20
Genetic material transfer	GB	**EVs**	Astrocytes	Tumor growth and maintenance	Ms, Hu	PDX, patient	NA	21,22
		**Cell fusion**	Neurons					
Hyperexcitability	Astrocytes	**Synaptogenesis**	GB	Increased GB invasiveness and connectivity, seizures	Ms, Hu	*In vitro*, PDX, patient	NA	23
Cx43		**Gap Junction**		Resistance to TMZ and VCR	Ms, Hu	PDX	Bentamapimod (AS602801, p-JNK inhibitor)	24, 25, 26, 27
Hypoxia	GB	**Tumor ECM**	Astrocytes	Reactive astrogliosis, tumor progression	Hu	PDX	Clinical imaging (Cu-ATSM probes)	28
WT1				Reactive astrogliosis	Hu	Patient	NA	29
Experimental genetic manipulation	Neurons		GB	Neoplastic dedifferentiation	Ms	*In vitro*, PDX	NA	30
	Astrocytes							
Brain injury		**Reactivity**		Tumorigenesis?	Hu	Patient	NA	31, 32, 33, 34
Brain irradiation		TG2		Tumorigenesis, MES transistion, tumor progression		*In vitro*, PDX, patient		31,35, 36, 37
GAP43		**Mitochondria transfer**		GB proliferation	Rt, Ms, Hu	Co-culture, PDX	NA	38
Glutamate	GB	**Tumor microtubes**	Neurons	Invasiveness	Ms, Hu	Co-culture, PDX, patient	Isoflurane, perampanel	39
NGLN3	Neurons	**Synapses**	GB	Progression	Ms, Hu	Co-culture, PDX, patient	ADAM10 inhibitor	40,41
Membrane depolarization		**Synapses, gap junctions**	OPC-like glioma	Tumor cell proliferation, patient brain hyperexcitability		Co-culture, PDX, patients	Meclofanamate, perampanel	42
TSP-1	GB	Ligand	Neurons	GB proliferation	Ms, Hu	Co-culture, PDX, patient	Gabapentin	43
TTYH1		**Neurites, synapses**		Axon outgrowth, tumor invasion	Hu	PDX, patient	NA	44
CA11/CA10	Neurons	Ligand	GB	*Decreased tumor growth*	Hu	PDX, patient	Prognosis marker	45
SOX10		**White matter**		*Pre-oligodendrocyte differenciation*	Ms, Hu	Co-culture, PDX	NA	46
Electrical activity		**Synapses**		Epileptiform neuronal hyperexcitability	Rt	Microelectrode arrays, *ex vivo*	NA	47
ACh/CHRM3				GB connectivity and invasion	Ms, Hu	Co-culture, PDX, patient	shRNAs, perampanel	48
GABA receptors		**Neurotransmitter**	LGG	Decreased tumor proliferation epileptic discharge	Hu	Patient	Bumetanide, sulfasalazine, valproic acid	49
			DMG/DIPG	Tumor progression	Ms, Hu	*In vitro*, PDX	NA	50
SEMA4F	Glioma	**Tumor ECM**	Neurons	Tumor progression and infiltration	Ms	PDX	NA	51
SCF/c-Kit	Neurons	Ligand	GB	Tumor angiogenesis	Ms, Hu	Co-culture, PDX, patient	Imatinib	52
NLGN-3		Ligand	OPG	Tumor formation	Ms	NF1-mutant mice	Light deprivation, ADAM10 inhibitor	53
BDNF/NTRK2		**Synapses**	DIPG	Glioma synaptic integration and plasticity	Ms, Hu	Co-culture, PDX	Entrectinib	54
tGLI1	BrBM	Unknown	Astrocytes	Increased metastatic potential	Ms, Hu	Co-culture, PDX	NA	55
proNGF	PrBM	**Axonogenesis**	Neuronal cell lines	Metastatic dissemination	Hu	Co-culture	proNGF immunoneutralization	56
MIF, IL-8, PAI-1	LuBM	Ligand	Astrocytes	Astrogliosis	Ms, Hu	Co-culture, PDX	NA	57
IL-6, TNF-α, IL-1β	Astrocytes	Ligand	LuBM	Proliferation	Ms, Hu	Co-culture, PDX	NA	57
MMP2/9		**Tumor ECM**	Lu/BrBM	MMPs mediated invasion	Rt, Ms, Hu	Co-culture, PDX	ONO-4817, marimastat, batimastat, MMP2, MMP3, and MMP9 immunoneutralization	58
Ach	Neurons	**Synapses**	LuBM	Metastatic progression	Hu, Ms	Co-culture, PDX	Carbachol, tetrodotoxin	59
IL-23	Astrocytes	Ligand	MelBM	Metastatic dissemination	Hu	Co-culture, patient	NA	60
CXCL10		Ligand		Migration, metastasis	Hu	Co-culture, PDX	CXCL10 Ab	61

Astrocytes, neurons, and neoplastic cells interact through paracrine signals or physical contacts (bold). For each element of language between two cell types, pro-tumoral (underlined) or antitumoral (italics) functions have been characterized in the associated studies.

Ab, antibody; Ach, acetylcholine; ADAM10, a disintegrin and metalloproteinase domain-containing protein 10; AMPA, α-amino-3-hydroxy-5-methyl-4-isoxazolepropionic acid; BDNF, brain-derived neurotrophic factor; BrBM, breast cancer brain metastases; c-KIT, tyrosine-protein kinase KIT; CA10/11, carbonic anhydrase-related protein 10/11; CHI3L1, chitinase-3-like protein 1; CHRM3, M3 muscarinic acetylcholine receptor; Cu-ATSM, diacetylbis(N(4)-methylthiosemicarbazonato) copper(II); Cx43, connexin 43/gap junction alpha-1 protein; DIPG, diffuse intrapontine glioma; DMG, diffuse midline glioma; ECM, extracellular matrix; EMT, epithelial-to-mesenchymal transition; EVs, extracellular vesicles; GAP43, growth-associated protein 43; GB, glioblastoma; GluN2B/NMDAR, GluN2B *N*-methyl-d-aspartate receptor; Hu, human; LGG, low grade glioma: LuBM, lung cancer brain metastases; MelBM, melanoma brain metastases; MES, mesenchymal; MGMT, O-methylguanine-DNA methyltransferase; MIF, macrophage migration inhibitory factor; MMP2/9/14, matrix metalloproteinase-2/-9/-14; Ms, mouse; NA, non applicable: NLGN-3, neuroligin-3; NF1, gene encoding the neurofibromin protein; NTRK2, neurotrophic receptor tyrosine kinase 2; OPC, oligodendrocyte precursor cell; OPG, optic pathway glioma; PAI-1, plasminogen activator inhibitor-1; PDX, patient-derived xenograft; p-JNK, phosphorylated Janus kinase; POSTN, periostin; PrBM, prostate cancer brain metastases; proNGF, nerve growth factor precursor; Rt, rat; SCF, stem cell factor; SEMA4F, semaphorin-4F; SOX10, transcription factor SOX10; SRGN, serglycin; TG2, transglutaminase 2; tGLI1, truncated glioma-associated oncogene homolog 1; TMZ, temozolomide; TNF-α, tumor necrosis factor alpha; TSP-1, thrombospondin 1; TTYH1, tweety family member 1; VCR, vincristine; *WT1*, Wilms tumor protein.

This review discusses the recent advances in the studies of the brain tumor–CNS interactome, including a guide for paracrine signaling with soluble factors and physical connections between the neoplastic cells and the brain parenchyma. This article focuses on the involvement of astrocytes and neurons in the context of GB and BM. Several excellent reviews focusing on other stromal cells such as brain microvascular endothelial cells^5^ or microglial cells^62^ are available elsewhere.

## Preface on Experimental Studies and Clinical Translatability

Experimental tools such as patient avatars (human cells implanted in immunocompromised laboratory animals) are classically used for preclinical brain tumor research. By numbers, these studies are the bulk of the literature gathered in this review. However, when dissecting the interactions between malignant and CNS cells, responsible models replacing mammalian organisms have emerged as a robust alternative. The fruit fly *Drosophila melanogaster* has been a central contributor to developmental biology, neuroscience, and brain cancer biology. Gene editing by either fusion (*FGFR3-TACC*) or constitutively active mutation (*EGFR-PI3K*) in *D. melanogaster*^63^ induced “fly gliomas” sharing histologic features with either lower (*FGFR3-TACC*) or higher (*EGFR-PI3K*) grade human tumors. For explorative biology, RNA interference screens in flies^64^ pinpointed genes responsible for fly egg cell polarization and migration. In patient transcriptomics databases, homologous genes exhibited matching roles during fly development and GB progression.

When considering cancer neuroscience studies, the *D. melanogaster* model facilitates *in situ* and *in vivo* glioma studies at the cellular resolution. For instance, the influence of GB cells on neuronal synapses density^65^ or GB neoplastic synapses formation^66^ has been studied in the fly. Fascinating chronotherapy studies in flies unveiled how glioma progression and associated degeneration of pacemaker neurons modify the circadian behavior of *Drosophila*.^66^ Forced re-synchronization of the light/dark phases significantly improved the outcome of tumor-bearing flies, providing new concepts to be explored for human therapy. Lastly, even smaller organisms such as the nematode worm *Caenorhabditis elegans* have been used for glioma therapy drug screens, further illustrating the value of alternative live models for brain tumor preclinical studies.^67^

## Tumor-Astrocyte Dialogue

**Figure 1:**
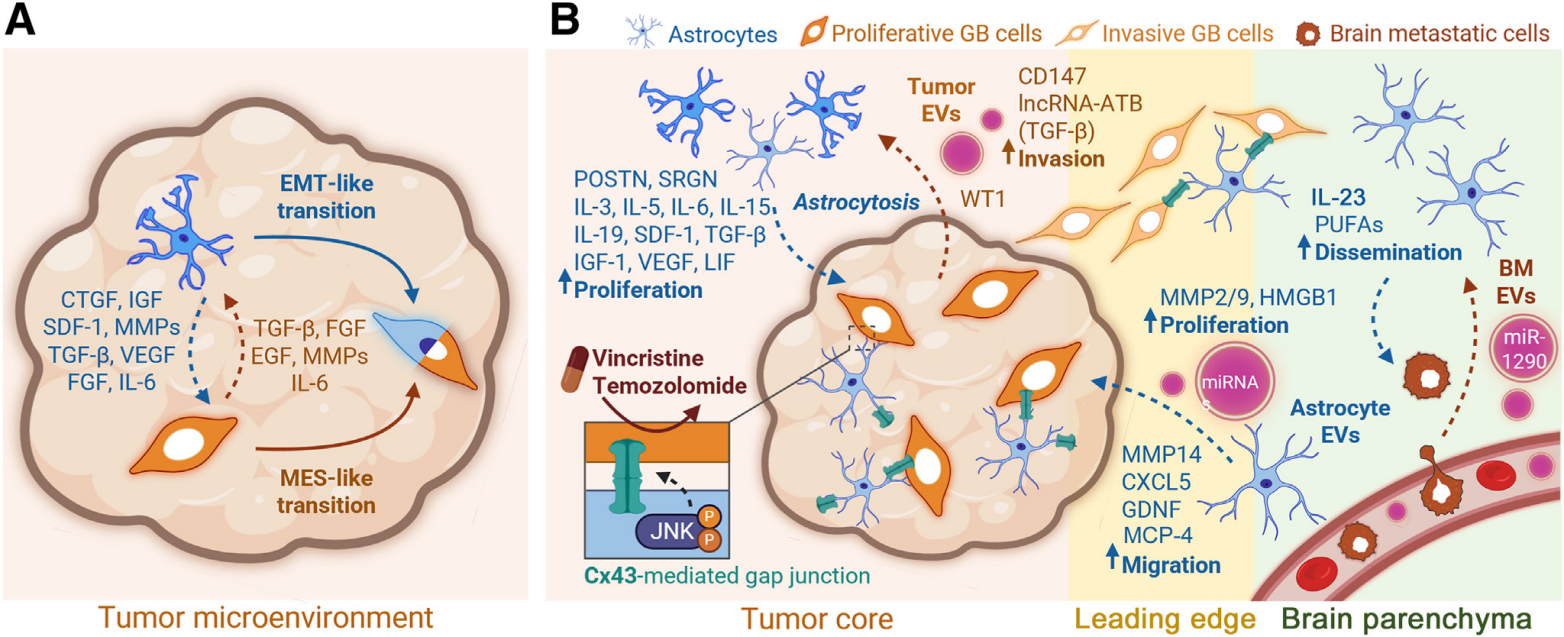
Brain tumor–astrocyte crosstalk modulating cancer progression. **A:** Astrocyte (blue) communication with glioblastoma (GB) cells in the tumor core (orange) includes paracrine signaling through chemokines, growth factors, or extracellular vesicles (EVs; purple), and physical interactions via gap junctions. Tumor-associated astrocytosis ignites GB progression via the release of interleukins and growth factors promoting tumor cell proliferation in the tumor core. For instance, astrocytic chemokines such as the monocyte chemoattractant protein-4 (MCP4), CXCL5, or the glial-derived neurotrophic factor (GDNF) have been shown to coordinate pro-invasive programs in GB cells at the leading edge. In turn, GB cells sustain astrocytosis by releasing, for example, tumor EVs containing reprogramming material such as long noncoding RNA (lncRNA) and miRNAs. In addition, GB microenvironment composition contributes to astrocyte reactivity through factors such as the Wilms tumor protein (WT1). Physical connections between GB cells and astrocytes through gap junctions enable direct exchange of biological material, including miRNA. Through these connexin 43 (Cx43)-mediated connections, reprogrammed tumor astrocytes provide shielding from therapies such as vincristine and temozolomide (bottom left insert). Naive/nonreactive astrocytes naturally release interleukins and polyunsaturated fatty acids (PUFAs), which have been shown to attract and support the extravasation of metastatic melanoma cells (dark brown) to the brain parenchyma. Similarly to GB cells, breast cancer brain metastatic cells (dark brown) are releasing EVs containing miRNAs (miR-1290), enabling remote reprogramming of astrocytes into tumor-supporting cells. **B:** The tumor core microenvironment fortifies epithelial to mesenchymal transition (EMT) in both astrocytic and tumor cell populations. Tumor core crosstalk between astrocytes and GB cells promoting astrocytic EMT include tumor sourced factors (orange) such the transforming growth factor beta (TGF-β), fibroblast growth factors (FGFs), epidermal growth factor (EGF), matrix metalloproteinases (MMPs) and IL-6. Similarly, astrocyte-originating molecules (blue) strengthen mesenchymal (MES)-like phenotypes in GB cells. Those include the connective tissue growth factor (CTGF), insulin-like growth factors (IGFs), stromal-derived growth factor-1 (SDF-1/CxCL12), MMPs, TGF-β, vascular endothelial growth factor B (VEGF), FGF, and IL-6. Astrocyte-tumor paracrine signaling functions as a loop, overcharging cancer aggressiveness. BM, brain metastases; HMGB1, high mobility group box 1; JNK, Janus kinase; LIF, leukemia inhibitory factor; lncRNA-ATB, long non-coding RNA activated by transforming growth factor β; POSTN, periostin; SRGN, serglycin.

Clinical observations of “bizarre astrocytes” within and surrounding neuropathologic lesions such as GB, amyotrophic lateral sclerosis, or gliosarcomas were first reported in the 1970s.^68^^,^^69^ The astrocytopathy was especially correlated with aggressive chemotherapeutic and radiotherapeutic regimens in patients.^70^ In the early 2000s, postmortem studies suggested that reactive astrocytes would provide a physical and chemical shield to brain tumors against immune system cell infiltration.^71^ The exact mechanism behind this shielding was first identified in 2019, when Heiland et al^72^ dissected the secretome of tumor-associated astrocytes. They identified several anti-inflammatory cytokines produced by the interaction between reactive astrocytes and microglia, including transforming growth factor-β, granulocyte colony-stimulating factor, and IL-10. Since then, accumulating evidence has shown how astrocytes can initially hamper the growth of brain neoplasms. Then, through reprogramming initiated by the tumor microenvironment, astrocytes ultimately exacerbate the tumor progression (Figure 1). Recent attempts at summarizing reactive astrocyte diversity have shed light on the complex relationships between astrocyte identity and anatomical location, age, sex,^73^ and pathologic microenvironments.^74^

### Soluble Factors

Epithelial-to-mesenchymal transition–like processes are observed both in GB and astrocytes, particularly at the tumor edge, induced by crosstalk between reactive astrocytes and GB cells^10^, ^11^, ^12^, ^13^, ^14^, ^15^, ^16^, ^17^, ^18^ (Figure 1A and Table 1). This transition, in addition to specific paracrine signaling, contributes to the aggressive progression of GB. Experiments of co-culturing astrocytes and GB cells revealed a transition to a mesenchymal state of the tumor cells, associated with accelerated progression and poor survival outcomes in preclinical models.^10^ Interestingly, evidence of GB–astrocyte paracrine signaling can be detected in meta-analyses of clinical samples with high astrocyte signature score from The Cancer Genome Atlas.^11^ In this study, authors identified GB overexpression of periostin (POSTN) and serglycin (SRGN), two secreted factors mediating astrocytic recruitment and activation (Figure 1A). In addition, periostin has been characterized as a biomarker for poor outcome in patients.^12^ Secreted by tumor-associated astrocytes, chitinase 3-like 1 (CHI3L1) binds to IL-13 receptor alpha 2 (IL-13Rα2) on the tumor cell surface. Activation of this CHI3L1–IL13Rα2 axis initiates the downstream mitogen-activated protein kinase and protein kinase B signaling pathways, consequently promoting GB cell proliferation.^13^ Globally, tumor-associated astrocytes secrete a range of factors known to accelerate tumor growth and stimulate GB cell invasion and extracellular matrix remodeling (Figure 1). For instance, human astrocytes secrete IL-6, which in turn up-regulate the expression of matrix metalloproteinase-14 (MMP14) in glioma cells. The IL-6–MMP14 axis plays a critical role in promoting glioma migration and invasion^14^ (Figure 1B).

*In vitro* studies underscore a significant increase in the migratory and invasive capabilities of glioma cells when co-cultured with normal human astrocytes. Elevated expression of IL-6 and MMP14 are strongly associated with reduced survival rates, particularly in high-grade gliomas.^15^ The mesenchymal state in GB reciprocally induces a reactive state in astrocytes and vice versa. This transition fosters therapy resistance, with IL-1β released by reactive astrocytes emerging as a key regulator in orchestrating a gradual mesenchymal transition. In addition, glioma-initiating cells undergo a transition to a reactive state exhibiting mesenchymal-like features and gene expression profiles akin to reactive astrocytes.^16^ To potentiate the release of these astrocyte-soluble factors in a paracrine loop manner, GB cells secrete transforming growth factor-β, fibroblast growth factor, epidermal growth factor, MMPs, and IL-6 (Figure 1). These secreted molecules from GB cells fuel astrogliosis, albeit with potential variations compared with the epithelial-to-mesenchymal transition as described for epithelial tumors^16^, ^17^, ^18^ (Figure 1A).

Interestingly, in addition to classical soluble factors and proteins, ion channels and ion transporters play a pivotal role in mediating communication between reactive astrocytes and GB cells. This interplay enhances tumor progression, metastasis, and tumorigenesis, while interference in this mode of communication might hold therapeutic potential. For instance, inhibition of ion channel– and ion transporter–mediated crosstalk has shown efficacy in impairing GB invasion and proliferation. Experimental combined therapy involving ion channel/ion transporter inhibitors with the therapy of reference, temozolomide, exhibited enhanced apoptosis of GB cells in both *in vivo* and *in vitro* settings^19^^,^^20^ (Figure 1B).

### Structural Communication

Recent studies have shed light on the critical involvement of extracellular vesicles (EVs) and gap junctions in mediating the bidirectional communication between astrocytes and GB. EVs are used by tumor cells as carriers for RNA, DNA, receptors, and proteins, including MMP2, MMP9, high mobility group box 1 (HMGB1), and CD147 (Figure 1B). When EVs undergo endocytosis by peritumoral stromal cells, they gradually induce neoplastic transformation of the microenvironment.

During the early stages of the tumor growth, astrocytes shelter the brain parenchyma from the tumor, notably through the re-uptake of glutamate to maintain homeostasis, delaying GB growth and migration.^21^ As the disease progresses, brain tumor EVs reprogram astrocytes into tumor-supporting cells, carrying resistance to temozolomide treatment. Those EVs have been shown to contain miRNAs, epidermal growth factor, fibroblast growth factor, IL-19, and colony-stimulating factor (Figure 1B). Remarkably, chemoresistance traits such as methylation status are carried by EVs under the form of O-methylguanine-DNA methyltransferase (MGMT) mRNA^14^^,^^15^^,^^18^^,^^22^ (Table 1).

Studies have shown an enrichment of genes promoting the formation of new neuronal synapses, a process called synaptogenesis, at the leading edge of gliomas. Synaptogenesis was associated with a distinct astrocyte population up-regulating genes controlling synapse formation and was previously characterized in other neuropathologies such as epilepsy. This synaptogenic effect is not ubiquitous to all astrocyte populations, further illustrating the great diversity of normal and tumor-supporting astrocytes.^23^

Gap junctions and physical contact between astrocytes and GB cells contribute to chemoresistance. This can be directly quantified from increased temozolomide-induced apoptosis in tumor cells expanded in monocultures compared with astrocyte tumor cell co-cultures.^37^ This chemoresistance is mediated by GB–astrocyte cell–cell contacts and the gap junction protein connexin 43 (Cx43)^24^, ^25^, ^26^, ^27^ (Figure 1B and Table 1). Gap junctions between GB and astrocytes promote tumor progression and chemoresistance, as well as increased Cx43 levels in patients, correlating with poorer prognosis. Experimental knockdown of astrocytic Cx43 reduced GB cell invasion *in vitro* and *ex vivo*, highlighting the contribution of astrocytes to the disease progression.^25^^,^^26^

Gap junction communication between GB and astrocytes can be pharmacologically inhibited by bentamapimod (AS602801, an experimental phosphorylated Janus kinase inhibitor), sensitizing GB to temozolomide and vincristine treatments^25^^,^^26^ (Table 1). Bentamapimod down-regulates expression of Cx43, potentially offering a strategy to overcome Cx43-mediated treatment resistance in GB^25^ (Figure 1B). Interestingly, bentamapimod also interrupted gap junction communication between lung cancer cells and astrocytes^27^ and was previously clinically assessed in humans against endometriosis progression (160 mg/kg twice a day for eight weeks; *https://clinicaltrials.gov*; NCT01630252), suggesting its potential for treatment of brain tumors.

Currently there is no reliable method to segregate “healthy” reactive astrocytes from neoplastic astrocytes, although several studies highlighted the potential of distinct astrogliosis markers. An earlier study investigated hypoxia, a prevalent feature in highly aggressive GB necessitating the development of diagnostic probes for clinical applications. Imaging probes such as radiolabeled diacethyl-bis(N4-methylthiosemicarbazone) typically accumulate in hypoxic regions of rat gliomas.^28^ Interestingly, an additional specific homing of this probe in the reactive astrogliosis delineating the tumor was identified. Locoregional uptake was associated with an up-regulation of copper transporters by the reactive glia, supporting the use of such tracers for *in situ* tumor profiling. Wilms tumor protein 1 (WT33) encoded by the *WT1* gene is another promising diagnostic candidate to improve astrocyte-based diagnosis of GB. *WT1* has been identified as aberrantly up-regulated in astrocytic tumor cells but not in the healthy brain or in nontumor-associated astrogliosis; this offers a unique opportunity to distinguish normal glia from neoplastic glia in patient samples.^29^

Gliomas can be experimentally generated from mature neurons and astrocyte cells through targeted genetic modification.^30^ Modifications of the microenvironment and/or introduction of exogenous factors during surgical resection of bulk tumors will exert a genetic stress on stromal cells, potentially promoting their transformation and contribution to relapses. Surgical intervention disrupts the tumor microenvironment, including astrocytic injuries leading to transcriptome and secretome modifications, promoting tumor proliferation and migration^10^ (Figure 1B). Reactive astrocytosis, which occurs in response to brain parenchymal injury, results in altered astrocyte functions, affecting homeostasis, neurogenesis, synaptogenesis, axon growth, the blood–brain barrier, and blood flow.

GB can be considered a form of brain damage that induces reactive astrocytosis, enhancing tumor growth and malignancy.^31^ Two separate reports show that traumatic brain injury increases the risk of brain tumor formation. A first report followed up 5000 patients with traumatic brain injury in Taiwan and compared them with 25,000 randomly selected enrollees.^32^ The result after 3 years of follow-up was a fivefold higher risk for malignancy after traumatic brain injury (6.28 versus 1.25 per 10,000). A follow-up study examined Afghanistan and Iraq war veterans with severe, penetrating, or moderate brain injury.^33^ These injuries were associated with increased risk of brain tumor.^32^^,^^33^

However, additional studies challenge the notion that significant brain injury increases the risk for developing malignant neoplasms. Analyses of individuals diagnosed with traumatic brain injury, cerebral ischemic infarction, and intracerebral hemorrhage revealed no increased risk of astrocytic neoplasms (eg, anaplastic astrocytomas, GB^1^) 5 years’ postinjury. Interestingly, a reduced long-term risk of developing malignant neoplasms in the brain injury group, compared with the normal population, was observed 20 years after injury.^34^ Extent of physical trauma might be the main factor driving tumorigenesis, as mild brain injury was not associated with increased tumor risk,^32^^,^^33^ explaining the discrepancies between studies focusing on brain injuries.

Accidental (or therapeutic) irradiation of the brain significantly increases stemness and radioresistance of gliomas. At the molecular level, irradiated astrocytes up-regulate transglutaminase 2 (TGM2), accelerating the mesenchymal transition and aggressiveness of GB.^35^^,^^36^ Moreover, spatial transcriptomics analyses of GB samples from irradiated patients identified GB cell reprogramming into an alternative phenotypic cell state. This cell state exhibited hybrid mesenchymal and astrocytic features with remarkable vascular co-option ability and radioresistance.^37^

Mitochondria transfer is a common mechanism in health and cancer. During neuron axon regeneration and astrocyte reactivity, mitochondria transit between cells occurs through intercellular connections facilitated by the growth-associated protein 43 (GAP43) (Figure 1). Mitochondria originating from astrocytes transfer to GB cells. Increased mitochondria numbers fortify GB cell respiration and up-regulate metabolic pathways linked to proliferation and tumorigenicity. Mitochondrial transfer from astrocytes to GB cells led to higher tumor cell dissemination and increased cancer-associated lethality in preclinical models^38^ (Table 1).

Decreased connections between tumor cells and astrocytes in favor of increased connectivity to neuronal networks increase GB invasiveness *in vivo*. Disconnected from astrocytes, invasive GB cells resemble neural progenitor–like cells and are sensitive to glutamatergic activity driving their migration and colonization.^39^

## Tumor–Neuron Dialogue

**Figure 2:**
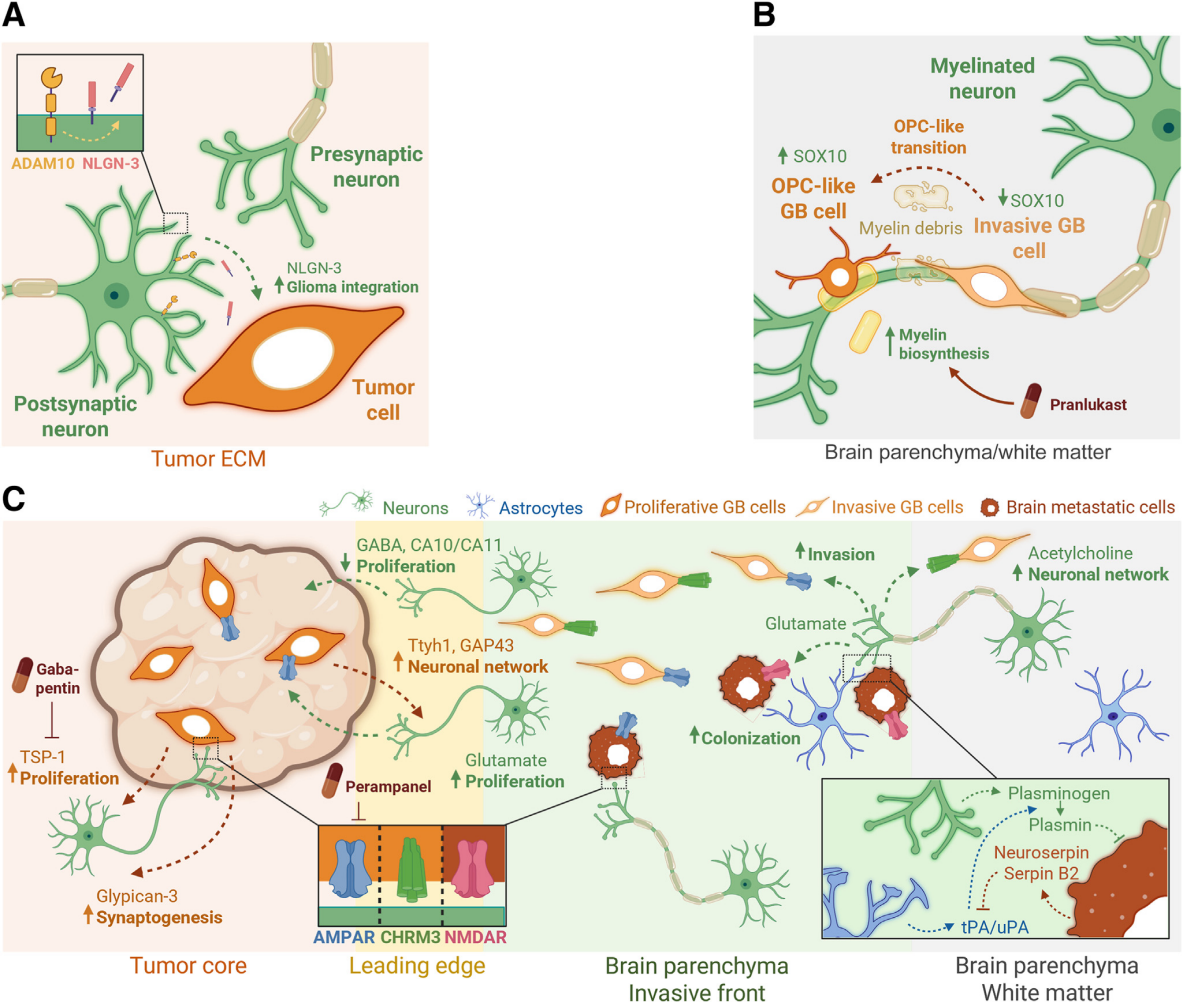
Brain tumor–neuron crosstalk modulating cancer progression. **A:** Glioblastoma (GB) cells are sensitive to neuronal synapse–specific neuroligin-3 (NLGN-3). Synapse formation and disconnection are coordinated by the active form of NLGN-3, cleaved from the membrane-bound immature form by a disintegrin and metalloproteinase domain-containing 10 (ADAM10; yellow). On GB cells, NLGN-3 will potentiate the formation of neoplastic synapse ersatz, neuronal-like differentiation, and neuronal network integration. **B:** GB invasion classically co-opts the preexisting brain structures such as microvascular endothelium and myelinated neuronal tracts of the white matter. Cell invasion partially degrades the extracellular matrix (ECM), releasing myelin-rich cell debris. On GB cells, myelin has been shown to induce a differentiation toward an oligodendrocyte precursor cell (OPC)-like cell state associated with up-regulation of the oligo-specific transcription factor SOX10. Interestingly, therapies increasing myelin production leverages OPC-like hysteresis, as GBs with oligo components are typically less malignant. **C:** Typical neuronal signaling pathways including neurotransmitters and electrochemical communication have been shown to support brain cancer progression in patients. Among the neurotransmitters modulating brain tumor progression, glutamate plays a central role in tumor cell integration to neuronal networks. Neuron-sourced glutamate binds to two ionotropic receptors expressed by brain tumor cells. Those include fast-acting (<1 millisecond) α-amino-3-hydroxy-5-methyl-4-isoxazolepropionic acid (AMPA; blue) and slower (>10 milliseconds) *N*-methyl-d-aspartate (NMDA; red) receptors, both modulating neuronal calcium activity. AMPA receptors are expressed in GB cells located at the tumor core and leading edge. In the core, higher glutamate concentrations activate AMPA receptor (AMPAR**)**-supporting tumor cell proliferation. At the invasive front, neuronal glutamate evokes AMPA signaling, facilitating GB cell integration to neighboring neurons through cellular microtubes. When fully integrated to neuronal networks, GB cells up-regulate a cholinergic receptor [M3 muscarinic acetylcholine receptor (CHRM3)] hence taking advantage of additional neurotransmission initiated by neuronal acetylcholine. NMDA receptors (NMDARs) are more specifically used by peripheral cancer cells progressing in the brain. Neuronal glutamate activates NMDARs expressed on synapses ersatz of brain metastatic cells. Interestingly, neurons can suppress brain tumor progression. Reduced tumorigenicity is mediated by the central inhibitory neurotransmitter GABA and the carbonic anhydrases 10 and 11 (CA10/11) produced by neurons of the leading edge. Once integrated to neuronal networks, glioma cells can modulate neuronal activity and network stability using membrane-bound proteins (**brown arrows**) such as tweety family member 1 (TTYH1) and growth-associated protein (GAP43). TTYH1 enhances axon outgrowth, and GAP43 promotes neoplastic network formation and GB cell proliferation and invasion. Similarly, tumor cells secrete glypican-3 promoting synaptogenesis and neural hyperexcitability. Brain metastatic cells integrate into tripartite-like synapses involving neurons and astrocytes (bottom right insert). Reactive astrocytes produce the urokinase/tissue plasminogen activator (uPA/tPA) converting plasminogen into plasmin. Plasmin is known to be tumor suppressive, used as neural defense mechanism against brain metastasis. Metastatic tumor cells block plasminogen activation by secreting the uPA/tPA inhibitors neuroserpin and serpin 2B. TSP-1, thrombospondin 1.

The complex relationship between neurons and GB cells has recently been identified as a key driver for tumor progression. This section explores various facets of neuronal signals and their involvement in GB growth, with potential new candidates for precision medicine. Key molecules promoting synaptogenesis and enhancing tumor growth^30^^,^^40^, ^41^, ^42^, ^43^, ^44^, ^45^, ^46^, ^47^, ^48^, ^49^, ^50^, ^51^, ^52^, ^53^, ^54^, ^59^^,^^75^, ^76^, ^77^, ^78^, ^79^, ^80^, ^81^, ^82^, ^83^ have been summarized in Figure 2 and Table 1.

### Soluble Factors

Glioma progression in the brain parenchyma takes advantage of neuronal proteins such as neuroligin-3 (NGLN-3). NGLNs are essential cell adhesion proteins during the formation of neuronal synapses. Synaptic stabilization is achieved through the interaction between presynaptic neurexins and postsynaptic NGLNs. NGLN-3 can be shed by disintegrin and metalloproteinase domain-containing protein 10 (ADAM10) proteolytic activity, which classically disconnects synapses^40^^,^^41^ (Figure 2A and Table 1). In brain cancer, soluble NGLN-3 binds to glioma cell surface (Figure 2A), activating focal adhesion kinase and the phosphatidylinositol 3-kinase/mammalian target of rapamycin pathway. Intracellular cascade activation induces up-regulation of NGLN-3 itself, as well as potassium channels classically expressed by neurons.^40^^,^^41^ Further studies from the same group revealed physical integration and bidirectional connectivity of GB cells to neuronal networks.^42^ The extent of GB integration to the neuronal networks can be detected in patients and provide readouts of the tumor progression.

Specific brain microenvironments reduce GB malignancy. Data from preclinical models and patient samples reveal that white matter fortifies oligodendrocyte precursor–like features in glioma cells. From the pathologist standpoint, gliomas with oligodendrocyte components are typically correlated with better response to therapy and longer survival in patients. From a mechanistic standpoint, oligodendrocytic transition is mediated by SOX10 (Figure 2B). This transcription factor is activated by myelin-associated proteins shed as GB cells co-opt to white matter tracts. An experimental induction of the oligo-like shift, which was induced via increased myelin production in the striatum with the cationic amphiphilic drug pranlukast, led to significantly prolonged survival in tumor-bearing mice.^46^

Electrocorticography techniques, the contact recording of electrical potentials at the surface of the exposed cerebral cortex, have recently been applied to patients with GB.^43^ In this study, a significant increase of the high-gamma band range power, associated with spikes of neuronal activity, was detected in patients with brain areas infiltrated with GB cells (Table 1). When dissecting the nature of these electrochemical changes in preclinical models, electrically active GB cells highly integrated in functionally connected brain areas (eg, with a synchronous increase in electrical activity) were found. The central molecule involved in this integration is the synaptogenic factor thrombospondin-1 (TSP-1) (Figure 2C), which is notably known for its involvement in neural circuit remodeling of the healthy brain.^43^ To disconnect neoplastic synapses in GB, the authors used the TSP-1 inhibitor gabapentin, an anticonvulsant approved by the US Food and Drug Administration for the treatment of epileptic seizures. Gabapentin treatment improved survival compared with control groups in preclinical studies. Additional synaptic markers such as Cx43, Gap43, and Ttyh1 have been identified as drivers of neuronal network formation, axon outgrowth, tumor invasion, and therapy resistance (Figure 2C). Similar to GB–astrocyte interactions, Cx43 and Gap43 facilitate interconnection and network formation among glioma cells, enhancing malignancy and resistance. Increased tumor cell connectivity further promotes cell invasion, proliferation, and axonal outgrowth^44^ (Figure 2C).

Recent developments in neuro-oncology have identified four emerging fields: electrochemical neural–cancer interactions, paracrine neural interactions, systemic neural interactions, and cancer therapy effects on the nervous system.^75^ However, not all neuronal paracrine signaling promotes GB progression (Figure 2C). For instance, carbonic anhydrase-related proteins 11 and 10 (CA10/CA11) are secreted neuronal synaptic proteins that function as neurexin ligands. These proteins have shown a negative correlation with glioma growth. Inhibition of *CA11* gene expression resulted in more aggressive tumor growth and reduced survival. CA10/CA11 secretion is regulated by the protein kinase B signaling pathway,^45^ and, in neuron GB co-cultures, CA10 inhibits glioma growth. Similarly, preclinical assessment for combination of glycogen synthase kinase 3 (GSK3) inhibitor (CHIR99021) and cAMP activator (forskolin) reported significantly reduced tumor growth. More specifically, this combination modulates neural crosstalk by directly affecting synaptic-like gene expression in GB cells, with moderate side effects for healthy neuronal networks, supporting clinical translatability.^76^

### Physical and Electrical Connections

When integrating to neuronal networks, glioma cells in culture or *ex vivo* disrupt the normal electrical activity of neurons^47^, ^48^, ^49^, ^50^, ^51^^,^^77^, ^78^, ^79^, ^80^ (Table 1). This leads to different electrochemical phenotypes, including epileptiform neuronal firing, or synchronized atypical short or long-lasting oscillations.^47^ Among neurotransmitters funneled by GB cells, glutamate plays a pivotal role in tumor proliferation and invasion (Figure 2C). Glioma cells express functional α-amino-3-hydroxy-5-methyl-4-isoxazolepropionic acid (AMPA) receptors, facilitating integration into glutamatergic neuronal networks. Remarkably, glutamatergic integration has been reported in breast cancer BM through the formation of tripartite synapses resembling astrocytes and hijacking *N*-methyl-d-aspartate (NMDA) receptors. Neural hyperactivity is mediated by potassium-dependent and AMPA receptor–driven mechanisms, forming electrically coupled networks via gap junctions in glioma cells. Targeting neural activity and gap junctions could influence growth modulated by neural activity.^42^^,^^77^ Once integrated, tumor cells trigger neural glutamatergic hyperexcitability, which in turn ignites tumor proliferation. Newly integrated proliferating glioma cells promote synaptogenesis through glypican-3 secretion, further reinforcing their relationship with the tumor stroma.

Tumor neuron hyperexcitability resembles the pathologic description of epilepsy. Tentative repurposing of epilepsy medication such as perampanel has shown promise for reducing GB-induced neuronal hyperexcitability^39^^,^^42^^,^^78^ (Table 1).

GB cell connectivity to neuronal networks further relies on M3 muscarinic acetylcholine receptors (CHRM3)^78^ (Figure 2C and Table 1). Using single-cell electrophysiology recordings and calcium imaging, the authors uncovered distinct connectivity profiles in GB cells, correlated with an expression “score” of synaptogenic markers *in silico*. They further implemented retrograde tracing, a classical neuroscientific methodology using fluorescent protein expression systems in engineered viruses, which propagates within physically connected cell networks.^78^ This enabled brain-wide connectivity assessments of live human GB cells in *in vitro*, *ex vivo*, and *in vivo* xenografts. Among identified neurotransmitters, dopaminergic, glutamatergic, and cholinergic elements of language were used by neurons to communicate with tumor cells (Figure 2C). Increased neuronal activity leads to acetylcholine release, supporting GB invasion and expansion in cortical brain areas. CHRM3 genetic (shRNAs) or pharmacologic (perampanel) interference effectively disconnected GB cells from neuronal networks and improved radiotherapeutic response in preclinical models.^78^

Progression of malignant GB is often associated with symptomatic epilepsy, once again attributed to neural hyperactivity surrounding the tumor stroma. Distinct elements in the glioma–neuron relationship contribute to symptomatic epilepsy, including different glutamate transporters such as the xCT system. Glutamate serves as a potent growth enhancer, whereas GABA, the central inhibitory neurotransmitter, counteracts this effect (Figure 2C). From a pharmacologic standpoint, understanding epilepsy in GB presents opportunities for novel therapeutic approaches, including approved epilepsy drugs such as sulfasalazine.^48^ Repeated anesthesia with isoflurane significantly reduces tumor invasion by affecting GB calcium activity. Isoflurane is currently used in the treatment of refractory status epilepticus, commonly known as persisting seizures in patients despite administration of first- and second-line medications. With validated indication for other neuropathologies,^49^ this supports isoflurane’s translatability to GB care when targeting tumor microtube formation.^8^^,^^39^

The contribution of GABA and its receptor GABA_A_ to glioma tumorigenesis and progression has been investigated in several studies, reporting different roles to this neurotransmitter. GABA_A_ is expressed by mouse and human GB cell lines, which also produce and release GABA. This auto/paracrine signaling has initially been shown to slow glioma proliferation mediated by the histone H2AX phosphorylation^79^ (Figure 2). *In* *vivo* pharmacologic studies show that GABA_A_ inhibition with bicuculline fortified tumor cell proliferation and tumor growth, whereas increasing GABA_A_ activity with muscimol prevented tumor initiation. More recently, studies of diffuse midline gliomas occurring in pediatric patients have found that GABA and GABA_A_ have an inverse role of promoting tumor growth and fortifying neoplastic synapses.^50^ Pharmacologic activation of GABA_A_ with lorazepam, an anxiety reliever given to younger patients with brain tumors undergoing neuroimaging, showed potentiated GABAergic currents in glioma, which fueled tumor cell proliferation and reduced survival in preclinical models.^50^ The authors speculate on the versatile role of GABA signaling based on the patient’s age (pediatric versus adult gliomas) and typical intertumoral heterogeneity observed in *IDH*–wild-type high-grade gliomas.

GB cell migration is influenced by electrotaxis, a response to electric fields increasing directed migration, albeit away from the tumor core in a symmetrical pattern. Pioglitazone, a peroxisome proliferator-activated receptor agonist, disrupts key signaling pathways, including epidermal growth factor receptor/phosphatidylinositol 3-kinase/protein kinase B, preventing electrotaxis-guided migration. Understanding these mechanisms may provide insights on how to clinically modulate and/or prevent GB dissemination supported by neuronal electrical activity.^80^ Contralateral brain activation promotes glioma infiltration and progression. Recent reports have identified the *SEMA4F* gene as being associated with brain network hyperactivity, correlated with enhanced glioma progression and infiltration.^51^ *In vivo* models show that overexpression of the SEMA4F in glioma cells leads to enhanced infiltration and shorter survival. On the other hand, glioma cell *SEMA4F* knockdown resulted in reduced infiltration and longer survival in preclinical studies.

## Connectivity during the Progression of Primary Brain Tumors and Peripheral BM

This last section highlights the relationships between brain microenvironment and GB phenotypic cell states. Although featured in fewer studies, the brain microenvironment is also a critical component for the progression of peripheral cancers to the CNS. We explore here the dynamic nature of these relationships and their implications for glioma and BM diagnoses with therapeutic opportunities.

### Complexity of GB Cell Architecture

In the *IDH*–wild-type GB, recurrent tumors tend to associate with neural, mesenchymal, and astrocytic phenotypes, whereas *IDH*-mutant GBs primarily consist of a proliferative phenotype enriched with stem cell–like tumor cells. The plasticity of *IDH* status, influenced by factors such as physical position and microenvironment (eg, leading edge or tumor necrosis), underscores the complexity of GB progression. Notably, recurrent GB cells exhibit a tendency to acquire neuron-like characteristics as RNA sequencing data suggest that approximately 66% of recurrent *IDH*-WT GBs associate with a neuronal phenotype. Neural signaling emerges as a key player in promoting invasiveness, exemplified by the expression of neurodevelopmental markers such as synaptome-associated protein 25 (SNAP25) at the invasive front of recurrent tumors, contributing to tumor cell plasticity.^52^

GB exhibits four cellular states: neural progenitor–like, oligodendrocyte precursor cell–like, astrocyte-like, and mesenchymal-like.^81^ Single GB tumors include all four states in different proportions, which reflects the dynamic influence of the tumor microenvironment and mirrors early brain development. Spatial transcriptional studies of human GB identified neurodevelopmental territories, enriched with gene expression signatures recapitulating oligodendrocytic lineages (pre-, early- and late-oligodendrocyte precursor cells), neural development, reactive immune, radial glia, and reactive hypoxia.^82^ All those are shaped by the tumor microenvironment influence on GB cellular states, illustrating the nuanced interplay between genetic and environmental factors. Notably, stress factors such as hypoxia drive the reactive hypoxia program, which emerges due to excessive proliferation, genetic alterations, and cellular migration toward nonhypoxic areas. Transcriptomic states in patient samples also correlate with pathologic features, including GB invasiveness and connectivity to neuronal networks.^48^ These findings highlight the potential for personalized GB treatment approaches by mapping out GB genetic transcriptional heterogeneity and dominant phenotypic states guiding the therapeutic decision.^82^ For instance, glioma infiltration disrupts brain hemodynamics and neurovascular structure, leading to seizures originating from the tumor margin and oxygen deficiency in the brain. Neurovascular-associated alterations due to tumor infiltration have been observed, shedding light on the intricate relationship between glioma and neural responses.^83^

Neurofibromatosis type 1 (*NF1*) gene mutations contribute to low-grade gliomas affecting the optic nerve (optic pathway gliomas) in early childhood. Light exposure has been shown to stimulate the development of optic pathway gliomas, with *NF1* mutation increasing NLGN3 shedding by ADAM10, and drives their progression. The findings emphasize the environmental and genetic factors contributing to the development of optic pathway glioma.^53^

Glioma synapses recruit mechanisms of adaptive plasticity. A recent study shows how glioma plasticity recapitulated neuronal plasticity established during memory formation in the adult brain.^54^ Binding of the brain-derived neurotrophic factor (BDNF), the endogenous ligand to the neurotrophic receptor kinase 2 (NTRK2), promotes the progression of diffuse intrapontine glioma through neuroplasticity mechanisms. This interaction increases the amplitude of glutamatergic current and neuron-to-glioma synapse formation. Interestingly, NTRK2 inhibitors such as entrectinib significantly reduced the proliferation of glioma cells *in vitro.* BDNF-NTRK2 signaling axis is a prime example of how glioma cells reinvent neural plasticity during tumor progression (Table 1).

Although there is no concrete evidence connecting brain physical trauma and GB incidence in patients (see *Structural Communication*), developmental studies underline the similarities between gliomagenesis and injury response.^84^ In this extensive transcriptomic study at the single-cell resolution of >100,000 mouse brain cells, the genetic evolution of preneoplastic, lower grade, and advanced brain tumors were compared to an experimentally induced brain injury. At early stages of both pathologies, a pre-neoplastic cell population type resembling neural crest cells is consistently captured. Those very undifferentiated cells are gradually replaced by neuronal precursor cell populations as both pathologies progress. These provocative findings not only propose that gliomagenesis and neurodevelopment share extremely similar cellular hierarchies but also that brain injuries could provide microenvironmental prerequisites for GB emergence.

### Molecular Intelligence of Peripheral Tumor Cells for Brain Microenvironment Integration

Although originating from very different milieu, malignant peripheral cancers frequently progress in the brain as BM. Indeed, BM are more frequent than GB^2^ yet experimental studies focusing on their integration to the brain are more limited. This can be explained in part by the lack of reliable experimental models and relevance to the human disease. BM are often occurring at a terminal stage of the peripheral progression when most therapeutic options have failed and lethality is high.^2^ Unfortunately, tumor sampling of BM is not systematically performed in clinics. However, recent clinical and academic collaborations have led to great advances in the data availability and accessibility of BM biology. Those recent examples generated and shared comprehensive data sets on the immune, vascular, and microenvironment landscapes of BM, such as the BrainTIME resource (Joyce Lab, *https://joycelab.shinyapps.io/braintime*, last accessed April 7, 2025). This dedicated section centralizes the key research studies reporting interconnectivity between neurons, astrocytes, and BM cells^55^, ^56^, ^57^, ^58^, ^59^, ^60^, ^61^ (Table 1).

Breast-to-brain metastasis represents a significant challenge, particularly in triple-negative and basal-type HER1-positive breast cancers. Breast cancer–derived brain metastatic cells have been shown to express neuronal NMDA receptors, enabling brain parenchyma colonization and reduced survival. Glutamate signaling emerges as a potent growth stimulator for breast-to-brain metastasis, leading to the formation of pseudo-tripartite synapses that mimic normal astrocyte–synapse interactions, rerouting a source of glutamate for tumor growth.^9^ Fast-progressing breast BM or tumor cells undergoing irradiation overexpress the truncated glioma-associated oncogene homolog 1 (tGLI1).^55^ This transcription factor modulates the expression of stem cell genes enabling breast BM adaptation, including manipulating neighboring astrocytes into tumor-supporting phenotypes.^55^

Central mechanisms such as tumor innervation are also observed in peripheral tumors, including prostate cancer. Cancer cells exploit nerve endings to modify the tumor microenvironment into a more supportive niche to promote growth. In cellular models, preventing this process using blocking antibodies targeting the precursor of the nerve growth factor offers potential for delaying cancer progression.^56^

Small-cell lung cancer is characterized by frequent widespread metastasis (up to two-thirds of patients), and survival postdiagnosis rarely exceeds 1 year. Active crosstalk between astrocytes and lung metastatic cells has been identified in preclinical models and in culture.^57^ Lung cancer cells release signals, including macrophage migration inhibitory factor (MIF), plasminogen activator inhibitor-1 (PAI-1), and IL-8, inducing astrocyte reactivity. In turn, tumor-activated astrocytes release IL-6, tumor necrosis factor alpha, and IL-1β promoting lung metastases growth.^57^ Tumor astrocytes also reshape the microenvironment of lung and breast cancer BM by releasing MMP2 and MMP9.^58^ Extracellular matrix modification by astrocytes triggers invasive dissemination of lung and breast BM in cultures and preclinical models, which can be counteracted by using pan-MMP inhibitors, including ONO-4817, marimastat, batimastat, or MMP2- and MMP9-blocking antibodies.^58^

Beyond molecular signals, BM from small-cell lung cancer are electrically active and evoke calcic responses.^59^ Similarly to GB cells, small-cell lung cancer–BM integration to neuronal networks is supported by the neurotransmitter acetylcholine. This integration can be prevented by using ion channel blockers such as the pufferfish poison tetrodotoxin. In addition, the authors showed that increased electrical stimulation of BM by neurons fueled tumor progression, whereas optogenetic and pharmacogenomic interference compromised small-cell lung cancer tumorigenicity *in vivo*.^59^

Melanoma BM exhibits distinct features compared with those of non-CNS metastases. In patients and preclinical models, cooperation between astrocytes and melanoma BM through signals such as IL-23^60^ and CXCL10^61^ have been shown to promote tumor cell dissemination in the brain. Single-cell RNA sequencing and transcriptomic analyses further identified intricate interactions between melanoma BM and the treatment-naive brain ecosystem.^85^ Notable changes include the activation of microglia and the promotion of chromosomal instability, neural-like differentiation, and shifts in gene signatures. For instance, neuronal-like differentiation of melanoma BM unlocked higher invasive capacity compared with parental cells, whereas overexpression of glucose metabolism, hypoxic response, and matrix proteins accelerated tumor growth. Non-brain metastasis, in contrast, displays a gene signature promoting epithelial-to-mesenchymal transition. These discoveries of BM-specific neural programs and ecosystem fitness after therapy provide new insights on BM progression and a new framework to design precision medicine targeting BM plasticity.^85^

## GB and BM Treatments from Preclinical to the Clinic

As mentioned in the opening of this article, the typical first-line treatment for newly diagnosed GB consists of maximal safe surgical resection followed by radiation therapy with concurrent and adjuvant temozolomide chemotherapy.^86^ Since 2018, tumor-treating fields can be prescribed to patients aged <65 years. Tumor-treating fields are low-intensity, 200 kHz alternating electric fields applied to the head of the patient, shown to reduce the tumor mitotic index and prolong survival up to 4.9 months.^87^ Over the past 2 decades, many attempts were made to repurpose peripheral cancer precision medicine for malignant brain tumors. These include drugs targeting DNA repair pathways, immunotherapies, vaccine therapies, oncolytic viruses, and modifications of radio/surgery protocols (reviewed elsewhere^88^). However, none of these strategies has significantly improved brain tumor patient survival.

The implementation of clinical trials focusing on GB is challenging due to the low incidence and progression speed of GB. To circumvent this scenario, it has been suggested that clinical studies could be performed as multicenter trials, which would increase enrollment numbers and improve accessibility for patients. In addition to clinical trial organization hurdles, individual patient heterogeneity makes it challenging to generalize therapeutic outcomes in brain tumors. For instance, highly innovative chimeric antigen receptor T-cell therapy usually hit the immunosuppressive wall built within the grade IV GB microenvironment. Interestingly, chimeric antigen receptor T-cell therapy shows promise for grade I to III pediatric gliomas in phase 1 trials,^89^ illustrating the diversity in chemosensitivity of glial-derived tumors.

Cancer neuroscience is still at its infancy in academia and *de facto* in the clinics. However, two standard-bearer trials are hopefully paving the way to the next generation of brain tumor precision medicine. First, the PerSurge (2 mg daily orally, 30 days prior to and 30 days after surgery; *https://euclinicaltrials.eu*; EUCT number 2023-503938-52-00) phase 2 trial takes advantage of α-amino-3-hydroxy-5-methyl-4-isoxazolepropionic acid receptor inhibition using the antiepileptic perampanel, which has been approved by the US Food and Drug Administration for the treatment of invasive GB. In preclinical assessments, perampanel disconnected tumor cells from neurons, impairing GB progression^8^^,^^44^ (Table 1). In the ongoing human trial, perampanel is administered before and after surgical resection.^90^ The second trial is currently investigating the inhibition of STAT3 in BM with silibinin (500 mg twice a day for a month, then once a day for 12 months; *https://clinicaltrials.gov*; NCT05793489). In preclinical models, the investigators of this clinical trial have shown that reshaping the brain astroglial and immune brain landscapes using silibinin together with immunotherapy has dramatically impaired the metastatic dissemination.^91^ This also extended the survival of mice from 20 to 35 days. In patients with BM, active phosphorylation of STAT3 in reactive astrocytes correlates with reduced survival. This ongoing human study therefore challenges two treatment options: whole-brain radiation therapy and silibinin versus whole-brain radiation therapy alone.

In conclusion, relationships between cancer cells and stroma are one of the hallmarks of malignancy. However, the degree of complexity of these interactions is unmatched to that of CNS tumors. Indeed, cancer cells implement both a molecular and electrochemical language to mimic, interact with, and reprogram the brain parenchyma. First, cooperation of primary brain tumor cells with astroglial populations is necessary for proper integration and propagation of the tumor. Following tumor progression, GBs and BMs reprogram astrocytes into cancer-promoting entities through physical connections and paracrine signaling. Supporting astrocytes exhibit distinct cell states and phenotypes associated with the peri- and intra-tumoral environment, accelerating the tumor progression. Astrogliosis at the tumor leading edge or in the resection cavity after neurosurgery has been shown to influence GB phenotypic cell states toward more aggressive and chemoresistant subtypes.

In addition to classical paracrine and autocrine routes leveraging growth factor signals, many brain tumors implement electrochemical connections that imitate neuronal synapses. Increasing connectivity by any means provides brain tumors with higher resistance to therapeutic interventions, from surgery to radiotherapies and chemotherapies. Remarkably, recent discoveries have uncovered how peripheral metastatic cancer progression to the brain also develops abilities to mimic neuronal networks and further infiltrate the parenchyma. This non-exhaustive compendium of the main elements of language between tumor and brain also reflect the recent advances in the field of cancer neurosciences. Additional research and review articles focusing on the bidirectional relationship between astrocyte and GB,^92^, ^93^, ^94^, ^95^, ^96^, ^97^ BM and neurons,^98^, ^99^, ^100^ as well as the consequence of brain tumor progression on the brain functionality,^101^, ^102^, ^103^ are compiled in Supplemental Table S1. This table provides the reader with additional insights on cancer neuroscience. In the context of clinical practice, a better understanding of the mechanisms regulating neuro/plastic features of brain tumors will define future therapeutic regimens aiming at disconnecting neoplastic networks. Recent experimental evidence has indeed shown how molecular excision of neoplastic synapses can isolate and make fragile tumors within the brain. This further improved the therapeutic response, even to traditional cancer chemotherapies.

## Disclosure Statement

None declared.
