# Supplementary material for: Deciphering the Dialogue between Brain Tumors, Neurons, and Astrocytes
Source: Am J Pathol. 2025 May 7;195(7):1193–208. doi: 10.1016/j.ajpath.2025.04.013 (PMC12264561; doi:10.1016/j.ajpath.2025.04.013)
Supplement: Supplemental Table S1 [file mmc1.docx]

**Supplementary Table 1: Extended literature including research, reviews, and editorial articles on brain cancer neuroscience.**

| Element of language | Source | Paracrine or **physical** connexion | Receiver | Deleterious or *beneficial* effect | Organism | Therapeutic application | References |
| --- | --- | --- | --- | --- | --- | --- | --- |
| HMGB1, miRNA | Astrocytes | **EVs, MVs** | GB | Invasion, proliferation | Ms, Hu | n/a | 92, 93, 94, 95, 96, 97 |
| IL-3, IL-5, IL-15 |  | Ligand |  |  |  |  |  |
| CTGF, IGF-1, SDF-1, MMPs, TGF-β, VEGF, FGF, IL-6 |  |  |  |  |  |  |  |
| CD147, lncRNA-ATB/TGF-β | GB | **EVs** | Astrocytes | Astrogliosis, invasion |  |  |  |
| IL-6, TGF-β, IGF-1, MCP-4, IL-19, VEGF, LIF |  | Ligand |  | EMT on reactive astrogliosis |  | n/a |  |
| mRNAs |  | **Gap Junctions** |  | Astrogliosis, migration |  |  |  |
| GDNF | Astrocytes | Ligand | GB | Migration |  |  |  |
| tPA/uPA |  |  | BrBM | *Decreased tumor co-option* |  | Immunotherapies, diagnosis via neurocognitive function assessment | 98 |
| plasminogen | Neurons |  |  |  |  |  |  |
| Neuroserpin, Serpin B2 | BrBM | Ligand | Astrocytes | Inhibits tPA and uPA, tumor progression |  |  |  |
|  |  |  | Neurons |  |  |  |  |
| GABA, glutamate, cysteine, AMPA, Ttyh1 | GB | **Microtubes** |  | Axon outgrowth, tumor invasion |  | n/a | 99, 100 |
| Epilepsy, neuronal activity | Neurons | Neurotransmitters | Gliomas | Brain functionality | Hu | Magneto-encephalography, *in vitro* | 101, 102, 103 |

Tumor astrocytes, neurons, and tumor cells interact through paracrine signals or physical contacts (bold). For each element of language between two cell types, pro-tumoral (underlined) or anti-tumoral functions (italics) have been characterized in the reported studies. Abbreviations: BrBM, breast cancer brain metastases, CD147, cluster of differentiation 147, CTGF, connective tissue growth factor, EMT, epithelial-to-mesenchymal transition, EVs, extracellular vesicles, FGF, fibroblast growth factor, GAP43, growth associated protein 43, GDNF, glial-derived growth factor, GB, glioblastoma, HMGB1, high-mobility group protein 1, Hu, human, IGF-1, insulin-like growth factor-1, IL-3, interleukin 3, IL-5, interleukin 5, IL-6, interleukin 6, IL-15, interleukin 15, IL-19, interleukin 19, LIF, leukemia inhibitory factor protein, MCP-4, macrophage chemoattractant protein-4, mi/lncRNA, micro/long non codant ribonucleic acid, MMP2/9, matrix metalloproteinase-2/9, Ms, mouse, tPA/uPA, tissue/urokinase plasminogen activator, SDF-1, stromal-derived growth factor-1, TGF-β, transforming growth factor beta, TTYH1, tweety family member 1, VEGF, vascular endothelial growth factor, xCT, cystine/glutamate antiporter
